# Erythromycin induced neuroprotection during prolonged deep hypothermic circulatory arrest in an acute porcine model

**DOI:** 10.1186/1749-8090-8-S1-O24

**Published:** 2013-09-11

**Authors:** C Koutsogiannidis, F Ampatzidou, O Ananiadou, K Diplaris, T Troupis, A Charchanti, G Drossos, E Johnson

**Affiliations:** 1 Cardiothoracic Surgery Department, General Hospital "G. Papanikolaou", Thessaloniki, Greece; 2 Department of Anatomy, University of Athens Medical School, Athens, Greece

## Background

The present study assesses whether preconditioning with erythromycin can improve neuronal viability in the neocortex following deep hypothermic circulatory arrest (DHCA) in the porcine model.

## Methods

Piglets were treated with erythromycin (25mg/kg, iv) (n=8) or vehicle (n=6) and subjected to 75 minutes of DHCA at 18°C, 12 hours after pretreatment. Three served as normal controls. After gradual rewarming, treatment animals were sacrificed and brains were perfusion-fixed and cryopreserved. Motor cortex was dissected from the left hemisphere and paraffin embedded for histologic staining with hematoxylin and eosin (HE). To assess neuronal damage, HE-stained paraffin sections (10μm) were examined by light microscopic examination at x400 magnification. Layer V of the motor cortex was counted. Neuronal injury was recorded when there was evidence of eosinophilic cytoplasm, cytoplasmic vacuolation, cell body shrinkage or nuclear pyknosis. Neuronal injury was scored on a scale of 0-5.

## Results

The peri-operative physiological variables did show significant variations with erythromycin drug treatment. The motor cortex from piglets pretreated with vehicle undergoing DHCA showed diffuse edema. Neurons showed a diffuse loss of Nissl substance, shrinkage of the perikaryon, and nuclear pyknosis with a mean neuronal injury score of 3.74 + 1.47. Neuronal injury in the motor cortex was significantly lower in animals pretreated with erythromycin (2.53 + 1.22; p < 0.01). Normal controls showed minimal neuronal injury (0.42 + 0.51).

## Conclusion

Pharmacologic preconditioning with erythromycin significantly improved neuronal viability in the motor neocortex of piglets undergoing HCA at 18°C. These findings suggest a potential clinical strategy of preemptive neuroprotection.

